# Effect of age on the efficacy and safety of clopidogrel plus aspirin vs aspirin alone in acute mild-to-moderate stroke: a secondary analysis of the ATAMIS trial

**DOI:** 10.3389/fneur.2025.1643517

**Published:** 2025-10-27

**Authors:** Hong-Ting Yan, Yu Cui, Zi-Ang Li, Ji-Ru Cai, Qi Wang, Xiao-Wen Hou, Hui-Sheng Chen

**Affiliations:** Department of Neurology, General Hospital of Northern Theater Command, Shenyang, China

**Keywords:** acute ischemic stroke, dual antiplatelet therapy, age, functional outcome, aspirin, clopidogrel

## Abstract

**Objectives:**

The association between age and the effect of antiplatelet therapy on clinical outcomes in patients with ischemic stroke remains unclear. This study aimed to explore the effect of age on the efficacy and safety of dual versus mono antiplatelet therapy in patients with mild-to-moderate ischemic stroke, based on the Antiplatelet Therapy in Acute Mild to Moderate Ischemic Stroke (ATAMIS) trial.

**Methods:**

This secondary analysis was based on the full analysis set from the ATAMIS trial. The primary outcome was an excellent functional outcome at 90 days, which was defined as a modified Rankin Scale (mRS) score of 0–1. The treatment effect of clopidogrel plus aspirin versus aspirin alone on outcomes was analyzed using age as either a categorical variable (age <65 vs. ≥65) or a continuous variable.

**Results:**

A total of 2,831 patients were included, with 1,249 in the subgroup aged <65 years and 1,582 in the subgroup aged ≥65 years. Compared to aspirin alone, clopidogrel plus aspirin significantly increased the likelihood of achieving an mRS score of 0–1 at 90 days in the ≥65-year age subgroup (adjusted OR, 0.77; 95% CI, 0.61–0.98; *p* = 0.031) but not in the <65-year age subgroup (78.02% vs. 78.62%, adjusted OR, 0.99, 95% CI, 0.75–1.32, *p* = 0.964). When age was analyzed as a continuous variable, the probability of an excellent functional outcome gradually decreased with advancing age in both treatment groups, but the decline was more pronounced in the aspirin-alone group, suggesting a relatively greater advantage of dual therapy among older patients.

**Conclusion:**

Our findings indicate that short-term dual antiplatelet therapy is associated with improved functional outcomes in patients aged ≥65 years but not in those aged <65 years. These findings suggest that age should be considered when developing antiplatelet strategies for treating acute mild-to-moderate ischemic stroke.

**Clinical trial registration:**

ClinicalTrials.gov, NCT02869009.

## Introduction

Current guidelines recommend reperfusion therapies as an effective strategy for patients with acute ischemic stroke (1). Nevertheless, a significant proportion of these patients are unable to receive reperfusion therapies due to strict therapeutic windows and limited access to endovascular interventions. Antiplatelet therapy is considered the gold standard for both acute management and secondary prevention of ischemic stroke (1–3). A multitude of randomized clinical trials has demonstrated that dual antiplatelet therapy is superior to mono antiplatelet therapy in mitigating the recurrence of ischemic stroke or transient ischemic attack (TIA) (2, 4–7).

As the global population ages, the increasing prevalence of ischemic stroke among older individuals poses a significant challenge for healthcare systems worldwide (8). Advanced age has been identified as an independent risk factor that contributes to poor post-stroke outcomes. This is largely due to complexities in drug reactions, increased risk of comorbidities, and a higher likelihood of adverse treatment effects (9–14). The management of ischemic stroke in older patients, defined as those aged 65 years and older, presents both a clinical challenge and a crucial area for research. Previous studies have shown that age affects the effectiveness of dual antiplatelet therapy, with older patients potentially exposed to a higher risk of bleeding and adverse events (15, 16). This increased risk can be attributed to their diminished metabolic and excretory functions, which may influence the blood concentrations of antiplatelet medications and increase the likelihood of hemorrhagic events (15). Therefore, it is essential to evaluate the associated risks and benefits for older patients before administering these drugs.

The Antiplatelet Therapy in Acute Mild to Moderate Ischemic Stroke (ATAMIS) trial was a randomized study that evaluated the efficacy and safety of dual antiplatelet therapy with clopidogrel and aspirin compared to aspirin alone in patients experiencing acute mild-to-moderate ischemic stroke [National Institutes of Health Stroke Scale (NIHSS) score 4–10] who presented within 48 h of symptom onset (17). The results of the ATAMIS trial demonstrated that clopidogrel plus aspirin was more effective than aspirin alone in reducing early neurological deterioration (END) at 7 days, while maintaining a similar safety profile in Chinese patients with acute mild-to-moderate ischemic stroke.

In this context, we performed the secondary analysis of the ATAMIS trial to investigate whether age affects the efficacy and safety of dual antiplatelet therapy in patients with acute mild-to-moderate ischemic stroke.

## Methods

### Study design and participants

This study is a *post hoc* analysis of the ATAMIS trial, performed in accordance with the STROBE guidelines. Detailed information about the design and protocol of the ATAMIS trial has been published (18). The ATAMIS trial was a multicenter, open-label, blinded-endpoint, randomized clinical trial that enrolled 3,065 patients between 20 December 2016 and 9 August 2022 to assess the efficacy of clopidogrel plus aspirin in patients with acute mild-to-moderate ischemic stroke who presented within 48 h of symptom onset. The follow-up was completed in October 2022.

Eligible patients were adults aged 18 years and older with acute ischemic stroke (AIS), who had a baseline NIHSS score of 4–10. The NIHSS scores range from 0 to 42, with higher scores indicating greater stroke severity. Patients were enrolled within 48 h of symptom onset. Patients who met the eligibility criteria for intravenous thrombolysis or endovascular treatment were considered to have received standard treatment and were therefore excluded from the trial. Other key exclusion criteria included the following: a clear indication for anticoagulation, a history of intracerebral hemorrhage, planned carotid revascularization, gastrointestinal or urinary tract bleeding within the past 3 months, and any allergy to clopidogrel or aspirin.

All procedures were performed according to the Declaration of Helsinki and approved by the ethics committee of the General Hospital of Northern Theater Command. Written informed consent was obtained from patients or their legally authorized representatives. The study was registered on ClinicalTrials.gov (NCT02869009).

### Procedures

The included patients were divided into two subgroups according to age: <65 years and ≥65 years. Each subgroup was further categorized into dual (clopidogrel plus aspirin) and mono (aspirin alone) antiplatelet groups. The patients in the dual antiplatelet group received a loading dose of clopidogrel 300 mg and aspirin 100 mg on the first day. From days 2–14, the patients were administered clopidogrel 75 mg and aspirin 100 mg per day. From days 15–90, they were administered clopidogrel 75 mg or aspirin 100 mg per day. The patients in the mono antiplatelet group received aspirin 100–300 mg daily from day 1 to day 14, followed by aspirin 100 mg daily from day 15 to day 90. All patients received stroke care based on guidelines for managing vascular risk factors, including the use of statins when deemed appropriate. Neurological status was assessed using the NIHSS score at baseline and on days 7 and 14 after randomization. Follow-up data were collected on day 7, day 14 (or at hospital discharge if earlier), and day 90 after randomization. Remote and on-site quality control monitoring and data verification were conducted throughout the study.

### Outcomes

In this *post hoc* analysis of the ATAMIS trial, the primary outcome was defined as an excellent functional outcome at 90 days. This is indicated by a modified Rankin Scale (mRS) score of 0 to 1 in patients with mild-to-moderate neurological deficits. The secondary outcomes included early neurological deterioration (END) at 7 days, defined as a >2-point increase in the NIHSS score compared to baseline (19) (excluding cerebral hemorrhage, hypoglycemia, cardiac complications, fever, and infection); change in the NIHSS score compared to baseline at 14 days; occurrence of new ischemic or hemorrhagic stroke within 90 days; and occurrence of other vascular events (pulmonary embolism, peripheral vascular event, or cardiovascular event) or death within 90 days (20). The adverse event outcomes included mucocutaneous hemorrhage, organ hemorrhage, or intracranial hemorrhage within 14 days and severe adverse events. A certified neurologist, who was blinded to the distribution of the groups, evaluated the follow-up NIHSS score. The final follow-up was conducted in person at 90 days. If an in-person visit was not feasible, a structured telephone interview was conducted by a trained and certified staff member at each center, who was blinded to the randomized treatment assignment. Central adjudication of the clinical outcomes and adverse events was performed by assessors who were unaware of treatment allocation and patient clinical details.

### Statistical analysis

The current study was based on the full analysis set from the ATAMIS study. Continuous variables were presented as median (interquartile range), while categorical variables were presented as frequency (percentage). The baseline characteristics were compared between the clopidogrel plus aspirin and aspirin alone groups based on whether the patient’s age was over 65 years using the Mann–Whitney U test for non-normally distributed continuous variables and the χ^2^ test for categorical variables.

Missing data for covariates included in the adjusted analyses were handled using simple imputation. Differences in the efficacy and safety outcomes were assessed using binary logistic regression models and generalized linear models (GLMs). Odds ratios (ORs) with 95% confidence intervals (CIs) were reported. The analyses were adjusted for prespecified prognostic factors, including sex, history of diabetes, history of hypertension, NIHSS score at randomization, time from symptom onset to initiation of antiplatelet treatment, and stroke etiology. If statistically significant differences were identified in the univariate analysis at baseline (*p* < 0.05), these variables were included as adjustment factors. Logistic regression models or GLMs with a multiplicative interaction term (age × treatment allocation) were used to test for an interaction effect of age (<65 years vs. ≥65 years) on the relationship between treatment (clopidogrel plus aspirin vs. aspirin alone) and outcomes. All tests were two-sided, and a *p* < 0.05 was considered statistically significant. All statistical analyses were performed using IBM SPSS software (version 25.0, SPSS Inc.).

## Results

**Figure 1:**
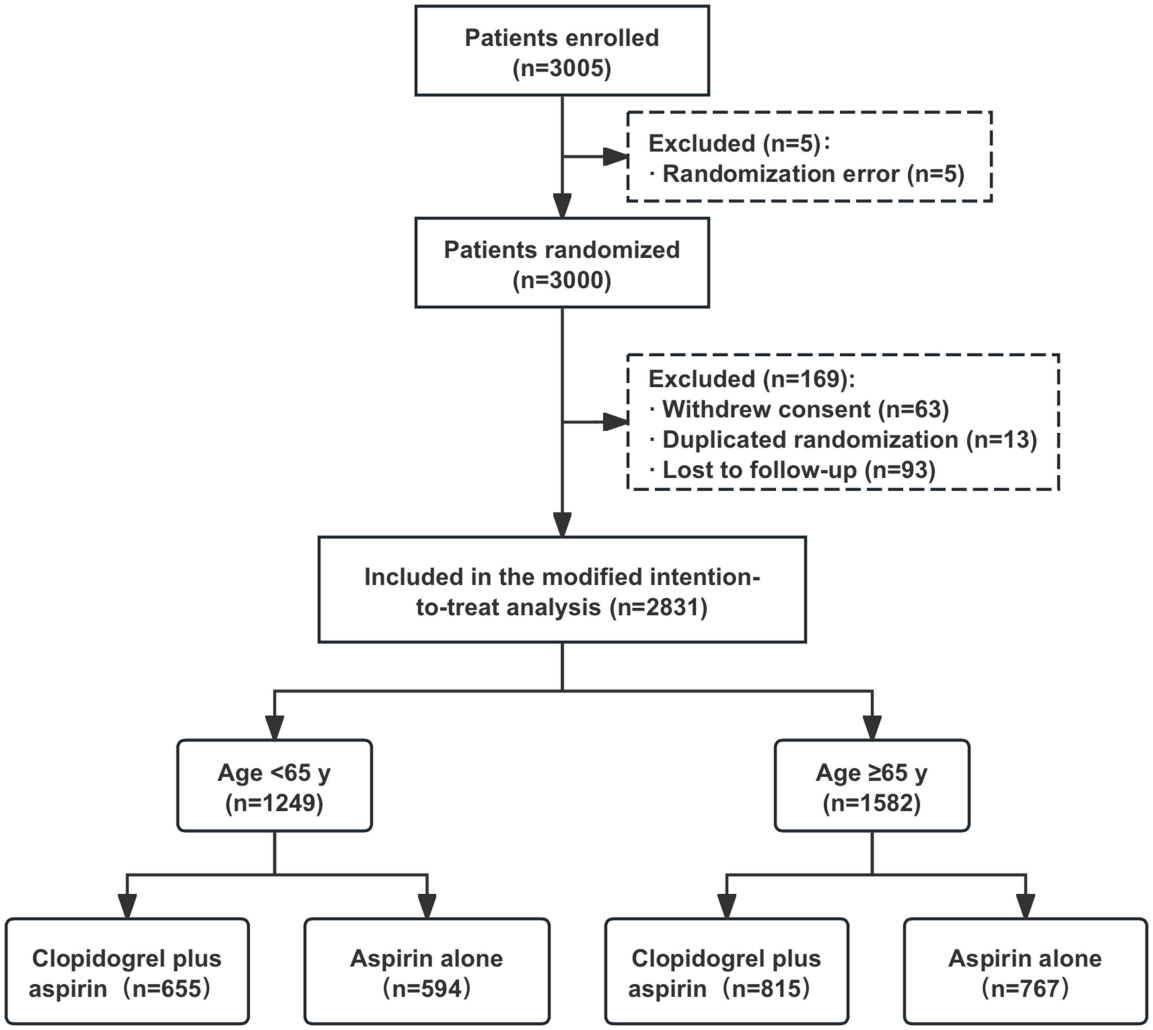
A flowchart of the patients.

After excluding 169 patients from the full analysis set of the ATAMIS study (comprising 63 patients who withdrew consent due to family disagreement and whose data were not used, 13 patients with duplicated randomizations, nine patients who were lost to follow-up at 7 days, and 84 patients who were lost to follow-up at 90 days), a total of 2,831 patients were included in the current study, with 1,249 in the < 65-year age subgroup and 1,582 in the ≥65-year age subgroup (Figure 1). In the <65-year age subgroup, there were 655 patients who received clopidogrel plus aspirin and 594 patients who received aspirin alone. In the ≥65-year age subgroup, there were 815 patients who received clopidogrel plus aspirin and 767 patients who received aspirin alone. As shown in Table 1, the baseline characteristics between the two treatment groups were well balanced across each age stratum (<65 years and ≥65 years).

**Table 1:** Baseline characteristics of the patients aged ≥65 years versus <65 years, stratified according to treatment arm.

Characteristic	Age <65 y			Age ≥65 y		
	Clopidogrel plus aspirin (*N* = 655)	Aspirin alone (*N* = 594)	*p*-value	Clopidogrel plus aspirin (*N* = 815)	Aspirin alone (*N* = 767)	*p*-value
Female	179/655 (27.33)	176/594 (29.63)	0.368	339/815 (41.6)	301/767 (39.24)	0.340
BMI	23.94 (22.31 ~ 25.53)	24.21 (22.31 ~ 25.81)	0.311	23.44 (21.97 ~ 25.06)	23.44 (21.72 ~ 25.39)	0.487
Current smoking	260/647 (40.19)	241/589 (40.92)	0.794	228/811 (28.11)	196/762 (25.72)	0.285
Current alcohol consumption	161/647 (24.88)	150/589 (25.47)	0.814	129/810 (15.93)	118/761 (15.51)	0.819
Comorbidities						
Hypertension	404/655 (61.68)	365/594 (61.45)	0.933	505/815 (61.96)	480/767 (62.58)	0.800
Diabetes	184/655 (28.09)	153/594 (25.76)	0.353	205/815 (25.15)	173/767 (22.56)	0.226
Previous ischemic stroke	185/647 (28.59)	174/590 (29.49)	0.728	283/812 (34.85)	266/767 (34.68)	0.943
Myocardial infarction	64/643 (9.95)	57/587 (9.71)	0.886	174/812 (21.43)	135/764 (17.67)	0.060
Atrial fibrillation	1/644 (0.16)	1/584 (0.17)	0.945	15/810 (1.85)	13/763 (1.7)	0.824
Hyperlipidemia	7/633 (1.11)	9/576 (1.56)	0.488	10/799 (1.25)	7/761 (0.92)	0.528
Previous TIA	4/652 (0.61)	1/586 (0.18)	0.220	2/810 (0.25)	2/764 (0.26)	0.953
OTT, hours	17 (8.23 ~ 26)	19.47 (8.5 ~ 26)	0.689	15.48 (6.75 ~ 26)	14.5 (7 ~ 26)	0.314
<24 h	390/655 (59.54)	346/594 (58.25)	0.643	489/815 (60)	473/767 (61.67)	0.497
≥24 h	265/655 (40.46)	248/594 (41.75)		326/815 (40)	294/767 (38.33)	
NIHSS score at randomization						
Median (IQR)	4 (4 ~ 6)	4 (4 ~ 6)	0.948	5 (4 ~ 6)	5 (4 ~ 6)	0.775
<7	585/655 (89.31)	532/594 (89.56)	0.886	720/815 (88.34)	683/767 (89.05)	0.658
≥7	70/655 (10.69)	62/594 (10.44)		95/815 (11.66)	84/767 (10.95)	
Estimated premorbid function (mRS score)						
0	462/655 (70.53)	427/594 (71.89)	0.599	573/815 (70.31)	551/767 (71.84)	0.711
1	193/655 (29.47)	167/594 (28.11)		240/815 (29.45)	215/767 (28.03)	
2	0	0		2/815 (0.25)	1/767 (0.13)	
Presumed stroke cause						
Large artery atherosclerosis	55/653 (8.42)	38/593 (6.41)	0.410	57/814 (7)	55/766 (7.18)	0.846
Cardioembolic	0	0		0	1/766 (0.13)	
Small artery occlusion	191/653 (29.25)	189/593 (31.87)		248/814 (30.47)	240/766 (31.33)	
Other determined cause	4/653 (0.61)	2/593 (0.34)		3/814 (0.37)	2/766 (0.26)	
Undetermined	403/653 (61.72)	364/593 (61.38)		506/814 (62.16)	468/766 (61.1)	
Location of the responsible vessel (identified by circulation infarction)						
Anterior	386/571 (67.6)	360/495 (72.73)	0.146	504/714 (70.59)	473/648 (72.99)	0.270
Posterior	169/571 (29.6)	120/495 (24.24)		190/714 (26.61)	151/648 (23.3)	
Anterior and posterior	16/571 (2.8)	15/495 (3.03)		20/714 (2.8)	24/648 (3.7)	
Time to hospital discharge, days	12 (10 ~ 14)	12 (10 ~ 14)	0.429	11 (10 ~ 13)	11 (9 ~ 13)	0.078

Data are shown as % (n/N) or median (IQR). BMI, indicates body mass index; TIA, transient ischemic attack; OTT, time from stroke onset to antiplatelet therapy; NIHSS, National Institutes of Health Stroke Scale; mRS, modified Rankin Scale. The presumed stroke cause was classified according to the Trial of ORG 10172 in Acute Stroke Treatment (TOAST) criteria, based on clinical findings, brain imaging, and laboratory tests. Scores on the modified Rankin Scale (mRS) for functional disability range from 0 (no symptoms) to 6 (death). A score of 0 indicates no symptoms, one indicates symptoms without disability, and two indicates mild disability.

**Figure 2:**
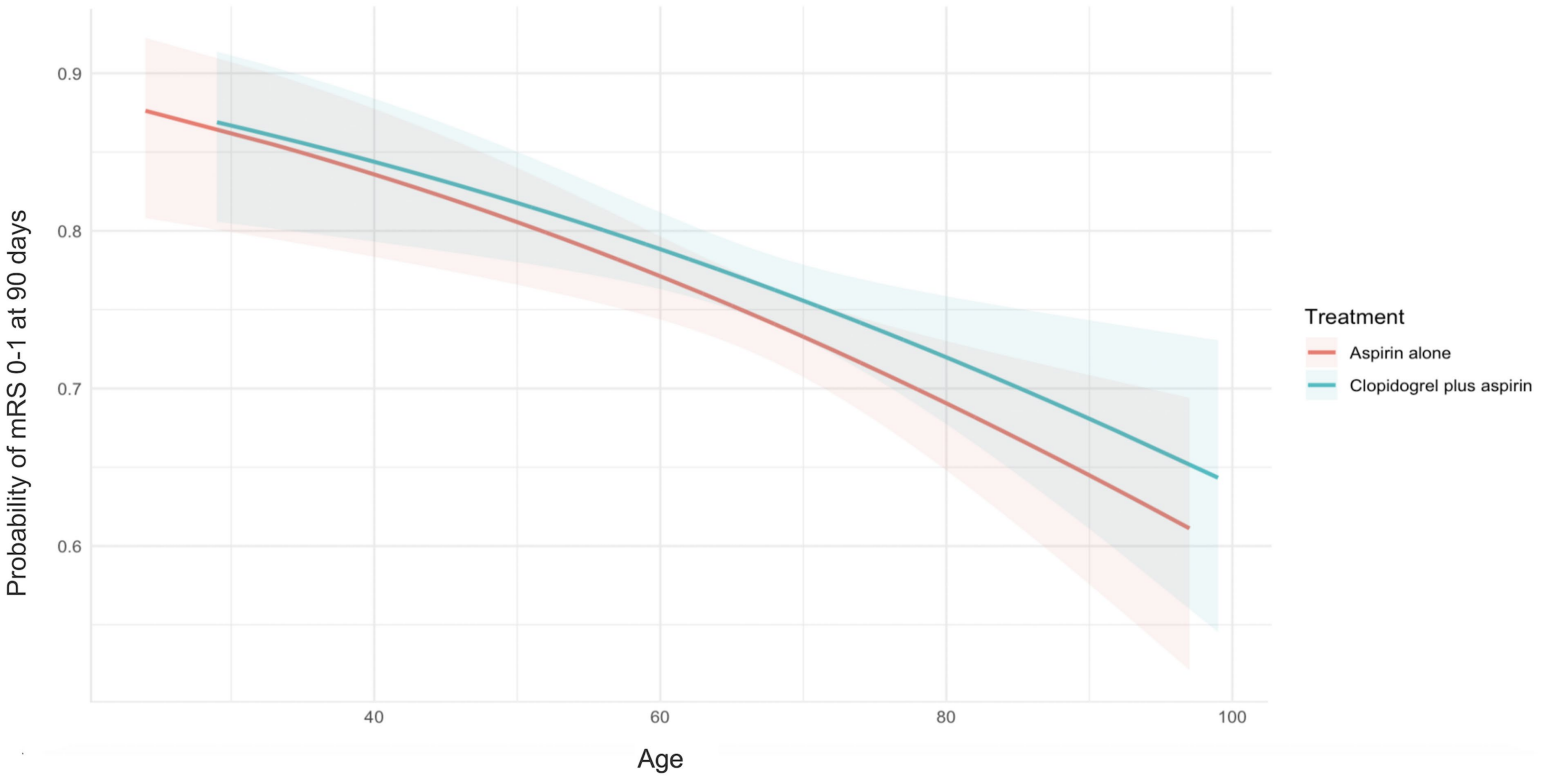
Probability curves for excellent functional outcomes at 90 days, stratified by treatment group. Increasing patient age was associated with a decreasing probability of excellent functional outcomes at 90 days in the clopidogrel plus aspirin group (OR, 0.98; 95% CI, 0.97–0.99) and the aspirin alone group (OR, 0.98; 95% CI, 0.97–0.99). No significant treatment-by-age interaction was observed (*p* = 0.813). The X-axis represents the patients’ age, and the Y-axis represents the probability of excellent functional outcomes at 90 days. The lines represent the best-fit probability curves, and the shaded areas represent their 95% CIs. The excellent functional outcome was defined as an mRS score of 0–1 at 90 days after randomization. CI, confidence intervals; mRS, modified Rankin Scale; OR, odds ratio.

**Figure 3:**
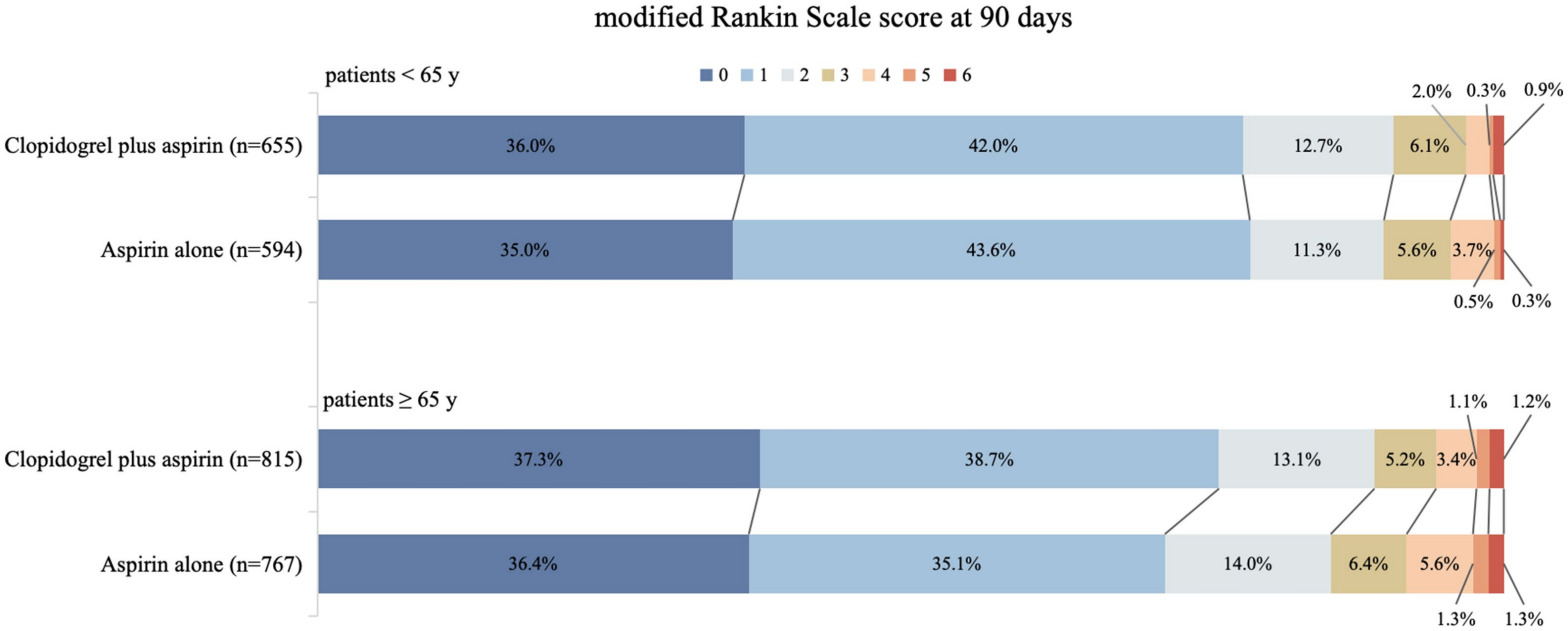
Distribution (in percentage) of modified Rankin Scale (mRS) scores at 90 days in the clopidogrel plus aspirin and aspirin alone groups. The scores ranged from 0 to 6, where 0 = no symptoms, 1 = symptoms without clinically significant disability, 2 = slight disability, 3 = moderate disability, 4 = moderately severe disability, 5 = severe disability, and 6 = death.

We detected the influence of age, treated as a continuous variable, on the treatment’s effect on achieving an excellent functional outcome. The probability of an mRS score of 0–1 at 90 days in the clopidogrel plus aspirin group (OR, 0.98; 95% CI, 0.97–0.99) was consistently higher than that in the aspirin alone group (OR, 0.98; 95% CI, 0.97–0.99) across all age ranges, but it decreased with increasing age in both groups (Figure 2). Table 2 presents the outcomes between the clopidogrel plus aspirin group and the aspirin alone group in the <65-year and ≥65-year age subgroups. The proportion of patients with an mRS score of 0–1 at 90 days in the clopidogrel plus aspirin group and aspirin alone group was 78.02 and 78.62% in the <65-year age subgroup. For patients in the ≥65-year age subgroup, the rates were 75.95% for those taking clopidogrel plus aspirin compared to 71.45% for those taking aspirin alone (Table 2 and Figure 3). Compared to aspirin alone, clopidogrel plus aspirin showed a numerically higher likelihood of achieving an mRS score of 0–1 at 90 days in the ≥65-year age subgroup (adjusted OR, 0.77; 95% CI, 0.61–0.98; *p* = 0.031). For the other secondary outcomes and safety outcomes, no significant differences were found between the treatment groups across any age subgroup. At the age threshold of 65 years, there was a significant interaction between clopidogrel plus aspirin and aspirin alone in terms of the mRS score of 0–1 at 90 days.

**Table 2:** Association of clopidogrel plus aspirin versus aspirin alone with clinical outcomes in the patients aged <65 years versus ≥65 years.

Outcomes	Age subgroups	No. (%) of events or median difference		Unadjusted		Adjusted		*P*_int_ value
		Clopidogrel plus aspirin	Aspirin alone	Treatment difference (95% CI)	*p*-value	Treatment difference (95% CI)	*p*-value	
Primary outcome								
mRS score of 0–1 at 90 days	<65 y	511/655 (78.02)	467/594 (78.62)	1.04 (0.79–1.36)	0.796	0.99 (0.75–1.32)	0.964	0.007*
	≥65 y	619/815 (75.95)	548/767 (71.45)	0.79 (0.63–0.99)	0.042*	0.77 (0.61–0.98)	0.031*	
Secondary outcomes								
END within 7 d	<65 y	27/655 (4.12)	34/594 (5.72)	1.41 (0.84–2.37)	0.192	1.57 (0.92–2.68)	0.095	0.248
	≥65 y	35/815 (4.29)	46/767 (6)	1.42 (0.91–2.23)	0.126	1.46 (0.93–2.31)	0.103	
Change in the NIHSS score at 14 d from baseline	<65 y	−0.56 (−1.1 ~ −0.32)	−0.51 (−0.93 ~ −0.25)	0.01 (−0.06 to 0.08)	0.744	0.02 (−0.05 to 0.09)	0.541	0.741
	≥65 y	−0.56 (−0.92 ~ −0.22)	−0.56 (−1.1 ~ −0.22)	−0.03 (−0.1 to 0.03)	0.297	−0.03 (−0.09 to 0.03)	0.355	
New stroke within 90 d	<65 y	4/655 (0.61)	4/594 (0.67)	1.1 (0.28–4.43)	0.890	1.11 (0.27–4.57)	0.891	0.904
	≥65 y	7/815 (0.86)	7/767 (0.91)	1.06 (0.37–3.05)	0.909	0.97 (0.33–2.85)	0.959	
Death within 90 d	<65 y	6/655 (0.92)	2/594 (0.34)	0.37 (0.07–1.82)	0.219	0.47 (0.09–2.48)	0.371	0.330
	≥65 y	10/815 (1.23)	10/767 (1.3)	1.06 (0.44–2.57)	0.891	1.11 (0.45–2.72)	0.817	
Safety outcomes								
Mucocutaneous hemorrhage	<65 y	1/655 (0.15)	0	NA	NA	NA	NA	NA
	≥65 y	1/815 (0.12)	1/767 (0.13)	1.06 (0.07–17.02)	0.996	1.25 (0.07–22.08)	0.877	
Organ hemorrhage	<65 y	1/655 (0.15)	1/594 (0.17)	1.1 (0.07–17.67)	0.945	1.18 (0.06–23.95)	0.915	NA
	≥65 y	1/815 (0.12)	0	NA	NA	NA	NA	
Intracranial hemorrhage	<65 y	1/655 (0.15)	1/594 (0.17)	1.1 (0.07–17.67)	0.945	1.22 (0.07–20.37)	0.891	NA
	≥65 y	0	1/767 (0.13)	NA	NA	NA	NA	
Symptomatic intracranial hemorrhage	<65 y	1/655 (0.15)	1/594 (0.17)	1.1 (0.07–17.67)	0.945	1.22 (0.07–20.37)	0.891	NA
	≥65 y	0	0	NA	NA	NA	NA	
Asymptomatic intracranial hemorrhage	<65 y	0	0	NA	NA	NA	NA	NA
	≥65 y	0	1/767 (0.13)	NA	NA	NA	NA	
Any bleeding events	<65 y	5/655 (0.76)	5/594 (0.84)	1.1 (0.32–3.83)	0.877	1.13 (0.32–3.93)	0.854	0.499
	≥65 y	2/815 (0.25)	5/767 (0.65)	2.67 (0.52–13.79)	0.242	2.61 (0.5–13.79)	0.258	
Adverse events	<65 y	58/655 (8.85)	56/594 (9.43)	1.07 (0.73–1.58)	0.726	1.08 (0.73–1.59)	0.703	0.881
	≥65 y	67/815 (8.22)	69/767 (9)	1.1 (0.78–1.57)	0.583	1.11 (0.78–1.57)	0.579	
Serious adverse events	<65 y	2/655 (0.31)	2/594 (0.34)	1.1 (0.16–7.86)	0.922	1.12 (0.16–8.06)	0.908	0.474
	≥65 y	6/815 (0.74)	2/767 (0.26)	0.35 (0.07–1.75)	0.202	0.39 (0.08–1.98)	0.256	

Data are shown as % (n/N) or median (IQR). mRS, modified Rankin Scale; END, early neurological deterioration; NIHSS, National Institutes of Health Stroke Scale. Pint value means the *p*-value for interaction. Adjusted for prespecified covariates, including sex, history of diabetes, history of hypertension, NIHSS score at randomization, time from symptom onset to antiplatelet treatment, and stroke etiology. Scores on the modified Rankin Scale (mRS) for functional disability range from 0 (no symptoms) to 6 (death). END was defined as an increase of two points or more in the NIHSS score between baseline and 7 days, excluding any changes attributed to cerebral hemorrhage, hypoglycemia, cardiac complications, fever, or infection. * Bold values indicate *p* < 0.05.

## Discussion

In this *post hoc* analysis of the ATAMIS study, we explored the effect of age on clinical outcomes in patients with acute mild-to-moderate ischemic stroke who received clopidogrel plus aspirin versus aspirin alone. The results showed that a higher proportion of excellent functional outcomes at 90 days was found in the clopidogrel plus aspirin group compared to the aspirin alone group in the ≥65-year age subgroup, while no significant differences were observed in patients aged <65 years. A significant interaction between age and treatment was observed (*p* = 0.007). Furthermore, when age was analyzed as a continuous variable, the probability of achieving excellent functional outcomes in the clopidogrel plus aspirin group gradually declined with advancing age; however, it remained consistently higher than that in the aspirin alone group across all age strata. Collectively, these findings suggested that age was associated with the effect of antiplatelet therapy on clinical outcomes among patients with acute mild-to-moderate ischemic stroke. Dual antiplatelet therapy may be associated with an increased probability of excellent functional outcomes in patients aged ≥65 years.

In the original ATAMIS trial, a significant reduction in END was found in the clopidogrel plus aspirin group compared to the aspirin alone group; however, the current study did not reproduce this finding in either subgroup. The discrepancy could be attributed to the reduced sample size and the number of events in each subgroup after stratification, which limited the statistical power to detect the difference. Furthermore, the benefit regarding excellent clinical outcomes disappeared in the patients aged <65 years. The inconsistency may be attributed to fewer bleeding events, recurrent strokes, and END due to lower stroke risk in this population, which makes it difficult to detect the benefit. Clinically, this suggests a very high probability of good outcomes in younger patients with aspirin, weakening the absolute benefit of dual antiplatelet therapy.

The CHANCE and POINT trials demonstrated that short-term (21–90 days) DAPT initiated within 24 h of stroke onset significantly reduced the risk of stroke in patients with minor ischemic stroke (3, 21), and the INSPIRES trial extended the benefit time window of DAPT to 72 h in particular patients with minor stroke. The SPS3 trial did not demonstrate the benefit of long-term DAPT in patients with lacunar stroke (22, 23). However, no study observed significant improvements in functional outcomes at 90 days. In the original ATAMIS study, clopidogrel plus aspirin was shown to reduce the incidence of early neurological deterioration compared to aspirin alone, but it was not found to improve functional outcomes in the overall population. The long-term benefits of dual antiplatelet therapy might vary depending on patient characteristics such as age and severity of atherosclerosis, whereas analyses of the overall population might mask the potential advantages in specific subgroups. This *post hoc* subgroup analysis aimed to investigate whether age influences the effect of dual antiplatelet therapy on functional outcomes at 90 days. Our study revealed that dual antiplatelet therapy significantly improved excellent functional outcomes at 90 days in acute mild-to-moderate ischemic stroke patients aged ≥65 years, whereas no significant difference was observed in patients aged <65 years. In the CHANCE, POINT, INSPIRES, and SPS3 trials, the treatment-by–age interaction was consistently non-significant; therefore, these studies rarely conducted deeper age-specific analyses. The discrepancy may be due to the difference in enrolled patient populations: patients with minor stroke in these trials versus patients with mild-to-moderate stroke in the ATAMIS trial.

In this study, this age-dependent difference might be partly explained by vascular and pharmacological factors. Older patients often have more complex and unstable atherosclerotic disease (24), and aspirin resistance is also reported more frequently in this group (25). Dual inhibition of thromboxane A2 and P2Y12 pathways might provide more complete platelet suppression in older patients compared to aspirin alone (26, 27). Diminished neural repair potential, together with a higher comorbidity burden, may place older patients at greater risk of adverse outcomes (28–32), which could explain why more intensive platelet inhibition offers additional benefits in this group. However, these potential mechanisms remain speculative and require confirmation in future clinical studies.

The primary strength of this study is that it is the first to investigate the effect of clopidogrel plus aspirin in acute mild-to-moderate ischemic stroke across different age strata, derived from a secondary analysis of the ATAMIS study. The benefit of DAPT on 90-day functional outcomes was identified in patients over 60 years of age. Nevertheless, we acknowledge that the present study has several limitations. Firstly, while the original ATAMIS trial demonstrated that dual therapy significantly reduced early neurological deterioration at 7 days (4.8% vs. 6.7%, *p* = 0.03), this *post hoc* analysis failed to reproduce this finding after age stratification, likely due to a reduced sample size and statistical power. Secondly, previous clinical trials have indicated that dual antiplatelet therapy might increase the risk of bleeding and intracranial hemorrhage in older patients compared to younger patients (15, 16). There was no significant difference in safety outcomes between the clopidogrel plus aspirin group and the aspirin alone group in this study, indicating acceptable safety profiles for dual antiplatelet therapy in older patients. However, given the reduced sample size due to subgroup analysis and the low event rate for safety outcomes, the safety profile in this population requires validation. In addition, despite adjusting for multiple confounders, unmeasured factors—such as CYP2C19 genotype polymorphism, which this study did not investigate but significantly influences clopidogrel metabolism and efficacy—may still persist. Furthermore, a potential limitation of our study is that patients aged ≥80 years were relatively few, which limited our ability to perform more refined subgroup analyses. Future studies with larger cohorts of very elderly patients are necessary to provide more reliable evidence on age-specific treatment effects. Finally, the current findings should be cautiously interpreted due to the nature of the *post hoc* analysis (33, 34).

In conclusion, this post hoc exploratory analysis of the ATAMIS trial suggested that short-term dual antiplatelet therapy is associated with improved functional outcomes in patients aged ≥65 years but not in those aged <65 years. These findings suggest that age may guide antiplatelet strategies in acute mild-to-moderate ischemic stroke.

## Data Availability

The raw data supporting the conclusions of this article will be made available by the authors, without undue reservation.

## References

[1] Kleindorfer DO Towfighi A Chaturvedi S Cockroft KM Gutierrez J Lombardi-Hill D. 2021 Guideline for the prevention of stroke in patients with stroke and transient ischemic attack: a guideline from the american heart association/American Stroke Association. Stroke. (2021) 52:e364–467. doi: 10.1161/STR.0000000000000375 34024117

[2] Hackam DG Spence JD. Antiplatelet therapy in ischemic stroke and transient ischemic attack. Stroke. (2019) 50:773–8. doi: 10.1161/STROKEAHA.118.023954, PMID: 30626286

[3] Johnston SC Easton JD Farrant M Barsan W Conwit RA Elm JJ. Clopidogrel and aspirin in acute ischemic stroke and high-risk tia. N Engl J Med. (2018) 379:215–25. doi: 10.1056/NEJMoa1800410, PMID: 29766750 PMC6193486

[4] Dong J Wang F Sundararajan S. Use of dual antiplatelet therapy following ischemic stroke. Stroke. (2020) 51:e78–80. doi: 10.1161/STROKEAHA.119.028400, PMID: 32264758

[5] Su Y Cheng X Dong Q. Dual antiplatelet therapy of clopidogrel and aspirin in secondary prevention of ischemic stroke: evidence and indications. CNS Neurosci Ther. (2015) 21:870–6. doi: 10.1111/cns.12419, PMID: 26122554 PMC6493159

[6] Bhatia K Jain V Aggarwal D Vaduganathan M Arora S Hussain Z. Dual antiplatelet therapy versus aspirin in patients with stroke or transient ischemic attack: Meta-analysis of randomized controlled trials. Stroke. (2021) 52:e217–23. doi: 10.1161/STROKEAHA.120.033033 33902301

[7] Shah J Liu S Yu W. Contemporary antiplatelet therapy for secondary stroke prevention: a narrative review of current literature and guidelines. Stroke Vasc Neurol. (2022) 7:406–14. doi: 10.1136/svn-2021-001166, PMID: 35393359 PMC9614124

[8] Tsao CW Aday AW Almarzooq ZI Anderson CAM Arora P Avery CL. Heart disease and stroke statistics-2023 update: a report from the American Heart Association. Circulation. (2023) 147:e93–e621. doi: 10.1161/CIR.0000000000001123, PMID: 36695182 PMC12135016

[9] Boehme AK Esenwa C Elkind MS. Stroke risk factors, genetics, and prevention. Circ Res. (2017) 120:472–95. doi: 10.1161/CIRCRESAHA.116.308398, PMID: 28154098 PMC5321635

[10] Wen SW Shim R Ho L Wanrooy BJ Srikhanta YN Prame Kumar K. Advanced age promotes colonic dysfunction and gut-derived lung infection after stroke. Aging Cell. (2019) 18:e12980. doi: 10.1111/acel.12980, PMID: 31199577 PMC6718525

[11] Ospel JM Kappelhof M Kashani N Menon BK Campbell BCV San Roman L. Effect of age and baseline aspects on outcomes in large-vessel occlusion stroke: results from the Hermes collaboration. J Neurointerv Surg. (2021) 13:790–3. doi: 10.1136/neurintsurg-2020-016621, PMID: 32929047

[12] Beuker C Köppe J Feld J Meyer CL Dröge P Ruhnke T. Association of age with 1-year outcome in patients with acute ischaemic stroke treated with thrombectomy: real-world analysis in 18 506 patients. J Neurol Neurosurg Psychiatry. (2023) 94:631–7. doi: 10.1136/jnnp-2022-330506, PMID: 37001983 PMC10359560

[13] Singer J Gustafson D Cummings C Egelko A Mlabasati J Conigliaro A. Independent ischemic stroke risk factors in older Americans: a systematic review. Aging. (2019) 11:3392–407. doi: 10.18632/aging.101987, PMID: 31127075 PMC6555455

[14] Van Horn N Kniep H Leischner H McDonough R Deb-Chatterji M Broocks G. Predictors of poor clinical outcome despite complete reperfusion in acute ischemic stroke patients. J Neurointerv Surg. (2021) 13:14–8. doi: 10.1136/neurintsurg-2020-015889, PMID: 32414889

[15] Wang D Gui L Dong Y Li H Li S Zheng H. Dual antiplatelet therapy may increase the risk of non-intracranial haemorrhage in patients with minor strokes: a subgroup analysis of the chance trial. Stroke Vasc Neurol. (2016) 1:29–36. doi: 10.1136/svn-2016-000008, PMID: 28959461 PMC5435197

[16] Zhang X Jing J Wang A Xie X Johnston SC Li H. Efficacy and safety of dual antiplatelet therapy in the elderly for stroke prevention: a subgroup analysis of the Chance-2 trial. Stroke Vasc Neurol. (2024) 9:541–50. doi: 10.1136/svn-2023-002450 38286485 PMC11732837

[17] Chen HS Cui Y Wang XH Ma Y-T Han J Duan Y-J. Clopidogrel plus aspirin vs aspirin alone in patients with acute mild to moderate stroke: the Atamis randomized clinical trial. JAMA Neurol. (2024) 81:450–60. doi: 10.1001/jamaneurol.2024.0146, PMID: 38466274 PMC10928538

[18] Xiaowen H Xiaoqiu L Xinhong W Chen H. Antiplatelet therapy in acute mild-moderate ischemic stroke (Atamis): a parallel, randomised, open-label, multicentre, prospective study. Stroke Vasc Neurol. (2019) 3:267–3. doi: 10.1136/svn-2018-000148 PMC6312068 30637134

[19] Xingyang Y Qiang Z Chun W Lin J Chai Z. Aspirin plus clopidogrel may reduce the risk of early neurologic deterioration in ischemic stroke patients carrying Cyp2C19*2 reduced-function alleles. J Neurol. (2018) 265:2396–403. doi: 10.1007/s00415-018-8998-1 30128710

[20] Ralph LS Scott EK Joseph PB Caplan LR Connors JJB Culebras A. An updated definition of stroke for the 21st century: a statement for healthcare professionals from the American Heart Association/American Stroke Association. Stroke. (2013) 44:2064–89. doi: 10.1161/STR.0b013e318296aeca 23652265 PMC11078537

[21] Wang Y Wang Y Zhao X Liu L Wang D Wang C. Clopidogrel with aspirin in acute minor stroke or transient ischemic attack. N Engl J Med. (2013) 369:11–9. doi: 10.1056/NEJMoa1215340 23803136

[22] Gao Y Chen W Pan Y Jing J Wang C Johnston SC. Dual antiplatelet treatment up to 72 hours after ischemic stroke. N Engl J Med. (2023) 389:2413–24. doi: 10.1056/NEJMoa2309137, PMID: 38157499

[23] Benavente OR Hart RG Mcclure LA Szychowski JM Coffey CS Pearce LA. Effects of clopidogrel added to aspirin in patients with recent lacunar stroke. N Engl J Med. (2012) 367:817–25. doi: 10.1056/NEJMoa1204133 22931315 PMC4067036

[24] Pelisek J Wendorff H Wendorff C Kuehnl A Eckstein HH. Age-associated changes in human carotid atherosclerotic plaques. Ann Med. (2016) 48:541–51. doi: 10.1080/07853890.2016.1204468, PMID: 27595161

[25] Morton M Kubiak-Balcerewicz K Sarnowska A Fiszer U. Biochemical aspirin resistance in acute stroke patients and its association with clinical factors: a prospective pilot study. Folia Neuropathol. (2021) 59:271–5. doi: 10.5114/fn.2021.109434, PMID: 34628792

[26] Li ZX Xiong Y Gu HQ Fisher M Xian Y Johnston SC. P2y12 inhibitors plus aspirin versus aspirin alone in patients with minor stroke or high-risk transient ischemic attack. Stroke. (2021) 52:2250–7. doi: 10.1161/STROKEAHA.120.033040, PMID: 34039032

[27] Chan NC Weitz JI. Antithrombotic agents. Circ Res. (2019) 124:426–36. doi: 10.1161/CIRCRESAHA.118.313155, PMID: 30702990

[28] Divya Thekkethala W Longstreth WT Jr Arnold AM Varadhan R Al Hazzouri AZ Cushman M. Factors associated with ischemic stroke survival and recovery in older adults. Stroke. (2017) 48:1818–26. doi: 10.1161/STROKEAHA.117.016726 28526765 PMC5553701

[29] Winstein CJ Stein J Arena R Bates B Cherney LR Cramer SC. Guidelines for adult stroke rehabilitation and recovery: a guideline for healthcare professionals from the American Heart Association/American Stroke Association. Stroke. (2016) 47:e98–e169. doi: 10.1161/STR.0000000000000098, PMID: 27145936

[30] Elizabeth RS Gail E Amy B. Executive function Poststroke: concepts, recovery, and interventions. Stroke. (2022) 54:20–9. doi: 10.1161/STROKEAHA.122.037946 36542071

[31] Nishioka S Wakabayashi H Nishioka E Yoshida T Mori N Watanabe R. Nutritional improvement correlates with recovery of activities of daily living among malnourished elderly stroke patients in the convalescent stage: a cross-sectional study. J Acad Nutr Diet. (2016) 116:837–43. doi: 10.1016/j.jand.2015.09.014, PMID: 27126155

[32] Engler-Chiurazzi EB Monaghan KL Wan ECK Ren X. Role of B cells and the aging brain in stroke recovery and treatment. Geroscience. (2020) 42:1199–216. doi: 10.1007/s11357-020-00242-9, PMID: 32767220 PMC7525651

[33] Han Y Lv HH Liu X Lv H‐H Dong Q Yang X‐L. Influence of genetic polymorphisms on clopidogrel response and clinical outcomes in patients with acute ischemic stroke Cyp2C19 genotype on clopidogrel response. CNS Neurosci Ther. (2015) 21:692–7. doi: 10.1111/cns.12426, PMID: 26177117 PMC6493114

[34] Xu J Wang A Wangqin R Mo J Chen Z Dai L. Efficacy of clopidogrel for stroke depends on Cyp2C19 genotype and risk profile. Ann Neurol. (2019) 86:419–26. doi: 10.1002/ana.25535, PMID: 31237713

